# The Meaning of a Master of Science Degree in Medical Physics

**DOI:** 10.1120/jacmp.v15i1.4729

**Published:** 2014-01-06

For our international readers, this issue's editorial is concerned with the ongoing saga of the meaning of the MS degree in medical physics within the United States. And with that statement, I probably just lost two‐thirds of my readers. Nevertheless, this is an issue that indirectly affects the international medical physics community, as the United States casts a long shadow. And for those of us within the US, we need to think carefully about the future of graduate medical physics education and speak honestly to our students who would honor us with their intention to join us as colleagues.

Most of us know the history all too well, and I will not bother to repeat it. 2014 has arrived, and medical physicists now will need to complete a CAMPEP‐accredited residency program in order to sit for the Board Certifications offered by the American Board of Radiology (ABR) in the various medical physics subfields. Many medical physicists have taken advantage of the closing door, and the number of new ABR certificates awarded in the past five years has been quite impressive; this is especially true in therapy physics. Job creation in our field predictably follows cancer incidence and steadily provides a market of 150‐175 new therapy physics jobs per year. This is not enough to employ all of the board‐certified therapy physicists looking for positions and the oversupply is estimated in the hundreds. Following 2014, we may see this oversupply diminish, as there will not be a sufficient number of residency program graduates to meet demand. As the residency programs expand, we may see a “soft landing”, as supply eventually balances demand a few years hence, but when we look around, we may not recognize the landscape.

The reason is there are four trends that are shaping the future of those who emerge from residency programs. And none of these trends favor those with a Master's degree in medical physics.

The first trend is the reality that students completing a CAMPEP PhD are better able to compete for residency positions. This is a simple statistical fact and well‐supported from the information we have from CAMPEP. This is also not surprising. My personal experience as director of a CAMPEP therapy physics program is that I looked at both MS and PhD students each year, and tried to hire the best individual. We hired MS and PhD students in almost equal numbers over the past ten years. This year, we have two extraordinary MS students. However, many programs prefer PhD candidates, perhaps valuing the additional research experience and perhaps appreciating candidates of greater diversity as many have MS degrees from other physics disciplines. It is likely the trend of PhD candidates successfully competing for residencies will continue.

The second trend is the increasing visibility and greater numbers of individuals with the PhD degree who apply through the alternative pathway. Many of our most capable medical physicists and many who have positions of significant responsibility within our profession are those from the alternative pathway. Program directors are becoming aware that these students are truly exceptional. Some highly prominent physics residency programs specialize in accepting only candidates from the alternative pathway. We should therefore expect these students, all of whom have completed a PhD, to be successful competing for residency slots.

The third trend is the creation of Doctor of Medical Physics (DMP) programs. The DMP combines an academic CAMPEP Master's degree with a CAMPEP residency program. The degree awarded is a professional doctorate. Today, there are almost ten programs either in existence, undergoing CAMPEP review, or being planned. Some programs are converting CAMPEP residencies to DMPs. The obvious rationale is to guarantee both the academic program and a residency to the candidate upon admission to the program. This should be much more attractive than an alternative offer to matriculate in a Master's program for two years without a guarantee the student will compete for a residency, commence the certification process, and enter the profession.

The fourth and final trend involves money. Many students are leaving undergraduate schools with crushing debt. Master's degree programs may mean even more debt. DMP programs may mean significant debt. There is usually some support for students completing a PhD program, but not all can make the monthly debt payments of an undergraduate/graduate education while pursuing a PhD, considering the limited support usually available.

The conclusion is sobering for those of you entering a Master's program in medical physics, and especially so if you are assuming student loans for this education. Those with Master's degrees are facing stiff competition and may be squeezed out of the marketplace for residencies. Student debt may prohibit you from entering a PhD program once you graduate. Finally, there is the possibility that CAMPEP residencies will become completely dominated by PhDs and DMPs. At some point the system may simply make it policy that the MS degree is not a sufficient credential to enter a residency, in order to give validity to the reality that exists. MS degrees in medical physics are, for practical purposes, not a sufficient credential to admit you to a CAMPEP residency program and the medical physics profession. There is a balancing trend, however. Those residency programs that do look carefully at CAMPEP Master's students will find that those candidates at the top of the pile are extraordinary individuals, indeed.

The conclusion is those who want to enter the profession should consider very carefully their opportunities and their finances. The meaning of the Master's degree in medical physics today is that it represents an opportunity — but not a guarantee — to become a professional medical physicist. And this opportunity must be weighed against your personal potential as a scientist and a professional, and your finances must be considered soberly. Medical physics is an extraordinary profession, but it comes with extraordinary complexity. And that is (and will be) the meaning of the Master's degree in medical physics in 2014.

Michael D. Mills, PhD

Editor‐in‐Chief

